# Retinal Detachment in Southwest Ethiopia: A Hospital Based Prospective Study

**DOI:** 10.1371/journal.pone.0075693

**Published:** 2013-09-27

**Authors:** Tsedeke Asaminew, Yeshigeta Gelaw, Sisay Bekele, Berhan Solomon

**Affiliations:** Department of Ophthalmology, College of Public Health and Medical Sciences, Jimma University, Jimma, Ethiopia; Medical University Graz, Austria

## Abstract

**Purpose:**

The incidence of retinal detachment in Blacks is generally considered to be low though there are few supporting studies in Africa. This study, thus, aimed at describing the clinical profile of patients with retinal detachment in Southwest Ethiopia.

**Methods:**

A hospital-based study was done on all consecutive retinal detachment patients who presented to Jimma University Hospital over six months period. A semi-structured questionnaire was used to collect patients’ sociodemographic characteristics and clinical history. Comprehensive anterior and posterior segment eye examinations were done and risk factors were sought for. Statistical tests were considered significant if *P* < 0.05.

**Results:**

A total of 94 eyes of 80 patients (1.5%) had retinal detachment (RD) and about 69% of patients were symptomatic for over a month before presentation. The mean age was 41.4 years (SD ±16.5). Fourteen patients (17.5%) had bilateral RD. At presentation, 61 eyes (64.9%) were blind from RD and 11 (13.8%) patients were bilaterally blind from RD. Rhegmatogenous RD was seen in 55 eyes (58.5%) and tractional RD in 22 eyes (23.4%). The most common risk factors were ocular trauma (32 eyes, 34.0%), myopia (23 eyes, 24.5%), posterior uveitis (13 eyes, 13.8%) and diabetic retinopathy (9 eyes, 9.6%). Most retinal breaks (25 eyes, 43.1%) were superotemporal and horse-shoe tear was the most common (19 eyes, 20.2%). Macula was off in 77 eyes (81.9%) and 38 eyes (69.1% of RRD eyes) had grade C proliferative vitreoretinopathy (PVR). Macular status was significantly associated with PVR (*P*=0.011), and duration of symptoms (RR=1.25, 95%CI: 1.059-1.475, *P*=0.040).

**Conclusions:**

A significant numbers of patients with ocular problem had retinal detachment, and nearly two third of the patients presented late. Trauma and myopia were the most important risk factors. People should be educated to improve their health seeking behavior and use eye safety precautions to prevent ocular trauma.

## Introduction

About 70% of global blindness is caused by cataract, trachoma and glaucoma [1]. In the National Survey on Blindness, Low Vision and Trachoma in Ethiopia [2], the major causes of blindness were reported to be cataract (49.9%), trachomatous corneal opacity (11.5%), refractive error (7.8%), other corneal opacity (7.8%), glaucoma (5.2%) and macular degeneration (4.8%).

Because of limited resources and the huge backlog of untreated cataract, trachoma and refractive error, retinal detachment (RD) and other retinal disorders in the developing countries seem to have a low priority. However, studies have shown that retinal diseases are accountable for blindness in a significant number of people. A population-based survey in India indicated that retinal diseases were the primary causes of blindness in a significant percentage (12.7%) of the studied population [3]. Though there is a general notion that black Africans have a low incidence of RD [4-9], there are evidences that RD might have been underestimated in Blacks. Foos examined the eyes of 2,334 subjects (322 of whom were African Americans) at post-mortem and found no racial variation in the age-corrected prevalence of lattice degeneration, retinal breaks or posterior vitreous detachment [10]. In a poor country, with limited facilities for the management of RD, patients may be less likely to attend an eye clinic than patients in a wealthy country, with better health care systems.

Population-based surveys on RD in developing countries are scarce and little is known about the incidence of retinal detachment in Africa. Because some of these studies are surgical case series, researcher could have excluded inoperable RD cases. A report from Luanda, Angola, indicated that, RD was the second most frequent cause of curable blindness after cataract [11]. A study with B-scan ultrasonography detected 71 RD cases in a 6-month period at Menelik II Hospital in Addis Ababa [12] and similarly a 5-year retrospective study at same Hospital reported 276 patients (305 eyes) with rhegmatogenous retinal detachment (RRD) [13].

Retinal detachment, especially RRD, is an ocular emergency that can cause a significant loss in vision but it is a treatable cause which requires a specialized care. The lack of adequate facilities for treatment of RD in developing countries makes the risk of RD related blindness relatively higher. The number of cataract extractions performed in these countries is also increasing; and this will compound the existing unaddressed problem as a result of post cataract surgery RD [14].

To our knowledge, there is no research done on retinal detachment in Southwest Ethiopia. Moreover, published literatures on retinal detachment in Ethiopia are not prospective and hence do not provide comprehensive data. This study, thus, aimed at describing the epidemiological and clinical profile of patients with retinal detachment and providing evidence for planning, designing preventive measures and care of RD patients in Southwest Ethiopia.

## Methods

### Ethics statement

The research was done in accordance with Declaration of Helsinki and Jimma University College of Public Health and Medical Sciences Institutional Review Board approved this study. Informed written consent was obtained from patients and/or guardians after explaining the purpose of the study using their local language. All patients with eye problems received the appropriate treatment and/or referred for better management.

We conducted a prospective hospital based cross-sectional study at the outpatient Eye Clinic of Jimma University Hospital Department of Ophthalmology (JUDO) from June 1, 2012-November 30, 2012. All patients who came to the hospital with any form of retinal detachment and who were able and willing to give informed consent were included in our study. Patients with any media opacity obscuring visualization of posterior segment of the eye were excluded.

We interviewed all retinal detachment patients (80 patients, 94 eyes) who fulfilled the inclusion criteria using a pretested semi-structured questionnaire for sociodemographic characteristics and clinical history, and did comprehensive anterior and posterior segment eye examinations with the same technique and instruments. We measured visual acuity with Snellen’s acuity charts and did objective refraction with autorefractor (Allergan, Humphrey, USA) and Heine Beta® 200 retinoscope (Heine, Germany). We did subjective refraction using trial lenses and recorded best corrected visual acuity. We performed pupillary reaction testing with a penlight, and external eye and anterior segment examinations with a Slit Lamp Biomicroscope (Zeiss, Germany). We dilated the pupils of all subjects with 1% tropicamide unless contraindicated and did detail posterior segment examination as much as the media clarity allowed. We also did stereoscopic examination of the disc and macula with a 90D and 78D Volk lens; a 20D lens was used for indirect ophthalmoscopy. Type of retinal detachment (rhegmatogenous, tractional and exudative) was determined; presence of retinal tears, holes, lattice degeneration, diabetic retinopathy or other anterior and posterior segment pathologies were documented. Macular status (on/off) was recorded. Proliferative vitreoretinopathy (PVR) was graded as A, B, C if present.

We edited and checked the data for consistency, and we then coded and entered the data into SPSS for windows version 16.0 (SPSS Inc., Chicago, IL, USA) for analysis. Association among variables was tested and considered statistically significant if *P* < 0.05.

## Results

Out of the total 5,310 patients seen at the outpatient Eye Clinic of JUDO in six month period (June 1-November 30, 2012), 94 eyes of 80 (1.5%) patients with retinal detachment were studied. The mean age of the patients was 41.4 (SD ±16.5) years while the median age was 42.5 with a range of 9-70 years. Male to female ratio was 2.6:1. Most patients were Oromo and Muslim accounting for 42.5% each. Around three quarters of the patients had at least primary education and 40% of the patients were farmers (Table 1). Patients came from far with an average distance of 87.5 km (SD±120.7km) from study setting and about 6% of them travelled more than 300km.

**Table 1:** Sociodemographic characteristics of retinal detachment patients.

	**Frequency (% from total)**		**Total**
**Characteristics**	**Male No (%)**	**Female No (%)**	**No (%)**
**Age**			
20-50 years	30 (37.5)	14 (17.5)	44 (55.0)
> 50 years	20 (25.0)	6 (7.5)	26 (32.5)
**Ethnicity**			
Oromo	22 (27.5)	12 (15.0)	34 (42.5)
Amhara	16 (20.0)	4 (5.0)	20 (25.0)
Kaffa	10 (17.2)	3 (3.8)	13 (16.2)
Dawro	4 (5.0)	3 (3.8)	7 (8.8)
Others	6 (7.5)	0 (0.0)	6 (7.5)
**Religion**			
Muslim	24 (30.0)	10 (12.5)	34 (42.5)
Orthodox	28 (35.0)	8 (10.0)	36 (45.0)
Protestant	4 (5.0)	3 (3.8)	7 (8.8)
Catholic	2 (2.5)	1 (1.2)	3 (3.8)
**Literacy**			
Illiterate	5 (6.2)	13 (16.2)	18 (22.5)
Primary school	42 (52.5)	4 (5.0)	46 (57.5)
Secondary school	8 (10.0)	4 (5.0)	12 (15.0)
Higher education	3 (3.8)	1 (1.2)	4 (5.0)
**Occupation**			
Farmer	29 (36.2)	3 (3.8)	32 (40.0)
Student	9 (11.2)	5 (6.2)	14 (17.5)
Housewife	0 (0.0)	11 (13.8)	11 (13.8)
Merchant	8 (10.0)	1 (1.2)	9 (11.2)
Daily laborer	4 (5.0)	2 (2.5)	6 (7.5)
Retired	4 (5.0)	0 (0.0)	4 (5.0)
Others	4 (5.0)	0 (0.0)	4 (5.0)
**Total**	**58 (72.5)**	**22 (27.5)**	**80 (100.0)**

Most patients, 60 (75.0%), came with a complaint of decreased vision only while others came with different complaints like flashes of light, floaters, shadows, redness, pain or these complaints with decreased vision (Table 2). Fifty five patients (68.8%) had one of these symptoms for at least one month before presentation (Table 3). At presentation, 61 eyes (64.9%) were blind from retinal detachment and 11 (13.8%) RD patients were bilaterally blind (Table 4).

**Table 2:** Presenting complaints of patients with retinal detachment.

**Presentation**	**No** (**%**)
Decreased vision only	60 (75.0)
Shadows	5 (6.2)
Flashes	1 (1.2)
Floaters	1 (1.2)
Decreased vision, flashes, shadows	10 (12.5)
Decreased vision, redness, pain	3 (3.8)
**Total**	**80 (100.0)**

**Table 3:** Duration of symptoms before presentation of patients with retinal detachment.

**Variable**	**Frequency** (**% within sex**)		**Total**
**Duration**	**Male No (%)**	**Female No (%)**	**No (%)**
< 1month	19 (32.8)	6 (27.3)	25 (31.2)
1-6 months	19 (32.8)	5 (22.7)	24 (30.0)
6 months -1year	15 (25.9)	4 (18.2)	19 (23.8)
> 1year	5 (8.5)	7 (31.8)	12 (15.0)
**Total**	**58 (100.0)**	**22 (100.0)**	**80 (100.0)**

**Table 4:** Best corrected visual acuity (BCVA) at presentation among patients with retinal detachment.

**Variable**	**Frequency** (**% from total eyes**)	
**BCVA**	**Eye with RD No (%)**	**Fellow eye with no RD No (%)**
6/6 - 6/18	4 (4.3%)	45 (47.9%)
< 6/18 - 6/60	4 (4.3%)	16 (17.0%)
< 6/60 - 3/60	6 (6.4%)	2 (2.1%)
< 3/60 - 1/60	19 (20.2%)	0 (0.0%)
< 1/60 - PL	48 (51.1%)	0 (0.0%)
NPL	13 (13.8%)	3 (3.2%)
**Total**	**94 (100%)**	**64 (68.1%)**

Abbreviations: RD, retinal detachment; PL, light perception; NLP, no light perception

**Figure 1:**
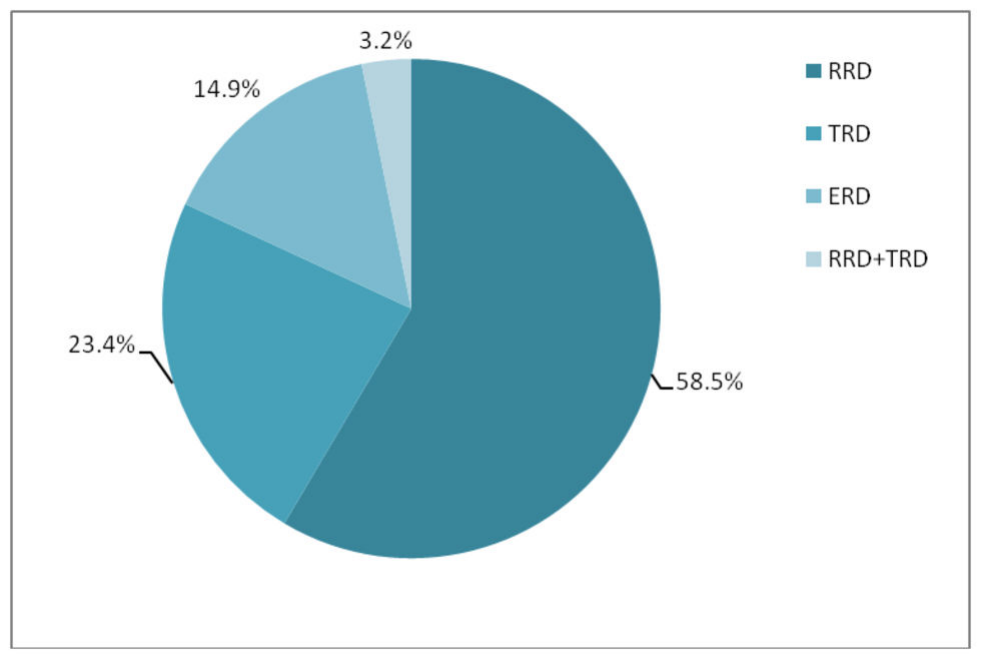
Types of retinal detachment (RD) among study patients. (RRD, rhegmatogenous RD; TRD, tractional RD; ERD, exudative RD).

Majority of the patients (82.5%) had RD only in one eye. The right eye only was affected in 23 patients (28.7%) while the left eye only was affected in 43 patients (53.8%). There were 14 patients (17.5%) with bilateral RD. Rhegmatogenous RD (RRD) was the commonest RD type (seen in 55 eyes, 58.5%) followed by tractional RD (TRD) (22 eyes, 23.4%) and exudative RD (ERD) (14 eyes, 14.9%) (Figure 1).

Risk factor was identified in 81 eyes (86.2%) and trauma was the commonest risk factor in the study setting. Patients gave history of trauma in 32 eyes (34.0% of total eyes) and in 25 eyes with RRD (45.5% of RRD eyes). Trauma was more common in male RD patients (66.6%) than in females (23.8%) but this was not statistically significant (*P* = 0.061).

Myopia of any severity was identified as a risk factor in 23 eyes (41.8% of RRD eyes). The mean power was -6.5 Diopters (D) for right eyes and -6.7D for left eyes. Seventeen (73.9% of myopic eyes) had ≥ -5D power. Of the myopic eyes with ≥ -5D, 14 (87.5% of ≥ -5D) had their macula off while of those < -5D only 4 eyes (66.7% < -5D) had macula off. However, there was no significant association between macular status and severity of myopia (*P* = 0.575).

Ten eyes (10.6%) had previous cataract surgery of which 7 (7.4%) were pseudophakic and the rest were aphakic. Of these, one had combined cataract surgery and trabeculectomy and another pseudophakic patient had Nd: YAG laser capsulotomy prior to the diagnosis of RD.

Diabetic retinopathy as a risk factor was found in nine eyes (9.6%) and all of these eyes had proliferative diabetic retinopathy (PDR) with TRD. Posterior uveitis was a risk factor for exudative detachments in 13 eyes (13.8%).

In some eyes, there were two or more risk factors. Lattice degeneration was identified in four (4.3%) eyes of which two also had myopia while another eye had trauma history. Snail track degeneration was found in one eye which had trauma history too. Some patients had systemic disease with either ocular manifestations or complications: one patient had Marfan syndrome with bilateral myopia and bilateral RRD; another patient had midfacial hypoplasia with bilateral myopia and bilateral RRD; and one patient had advanced breast cancer and presented with bilateral exudative RD.

**Figure 2:**
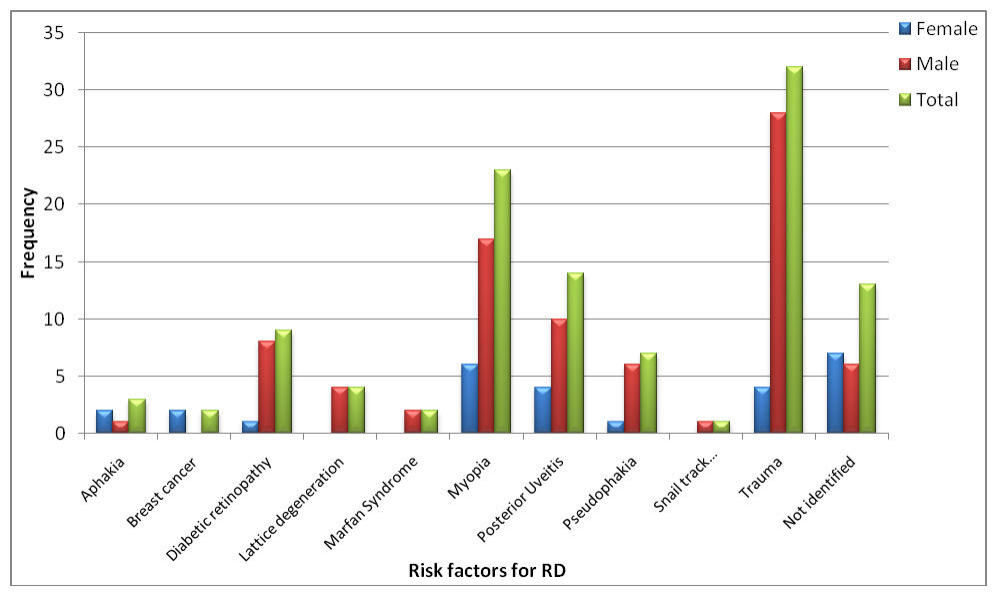
Risk factors of retinal detachment (RD) in eyes of the study patients.

The commonest risk factor for RRD in females was myopia, six (40.0% of eyes of females with RRD) while in males it was trauma, 21(52.5% of eyes of males with RRD) (Figure 2). But there was no statistically significant association among the risk factors and sex (*P* = 0.492).

Retinal breaks were found in 50 eyes (86.2% of eyes with RRD and combined RRD & TRD). In 33 eyes (56.9% of eyes with RRD and combined RRD & TRD) only one retinal break was identified. Two breaks were found in ten eyes (17.2% of eyes with RRD and combined RRD & TRD). Three breaks were found in five eyes (8.6% of eyes with RRD and combined RRD & TRD). Two eyes had four breaks (3.4% of eyes with RRD and combined RRD & TRD). In the remaining eight eyes (13.8%) retinal break could not be identified.

The commonest location of retinal breaks was superotemporal (ST) occurring in 25 eyes (26.6% of total eyes, 43.1% of RRD and combined RRD & TRD eyes). Superotemporal breaks were common among males (18 eyes, 45.0% eyes of males) while inferotemporal (IT) breaks were common among females (5 eyes, 33.3% eyes of females). There was, however, no statistically significant association between sex and location of break (Table 5).

**Table 5:** Locations of retinal break in eyes with rhegmatogenous retinal detachment.

**Variable**	**Frequency (% within sex)**		**Total(% from RRD eyes)**
**Location of retinal break**	**Male No (%)**	**Female No (%)**	**No** (**%**)
Not identified	7 (17.5)	0 (0.0)	7 (12.7)
Superotemporal	18 (45.0)	4 (26.7)	22 (40.0)
Superonasal	2 (5.0%)	1 (6.7%)	3 (5.5%)
Inferotemporal	5 (12.5%)	5 (33.3%)	10 (18.2%)
Inferonasal	0 (0.0%)	1 (6.7%)	1 (1.8%)
Macula	3 (7.5%)	3 (20.0%)	6 (10.9%)
Superotemporal + macula	5 (12.5%)	1 (6.7%)	6 (10.9%)
**Total**	**40 (100.0%)**	**15 (100.0%)**	**55 (100.0%)**

Abbreviation: RRD, rhegmatogenous retinal detachment

Twelve eyes (12.8% of total eyes and 20.7% of RRD and combined RRD & TRD eyes) had macular holes of which 6 (6.4% of total eyes and 40.3% of RRD and combined RRD & TRD eyes) had another break at the superotemporal area (Table 5).

The commonest type of retinal break was U-tear (horse-shoe) occurring in 19 eyes (20.2%) and the least common being dialyses occurring in three eyes (3.2%) of the total and 5.2% of RRD and combined RRD & TRD eyes (Table 6).

**Table 6:** Type of retinal break among eyes with retinal detachment.

**Variable**	**Frequency** (**% within sex**)		**Total**
**Type of retinal break**	**Male No (%)**	**Female No (%)**	**No (%)**
Not identified	31 (45.6%)	11 (42.3%)	42 (44.7%)
U-tear	12 (17.6%)	7 (26.9%)	19 (20.2%)
Operculated	3 (4.4%)	0 (0.0%)	3 (3.2%)
Atrophic hole (peripheral) only	4 (5.9%)	1 (3.8%)	5 (5.3%)
Macular hole	3 (4.4%)	3 (11.5%)	6 (6.4%)
Giant tear	2 (2.9%)	1 (3.8%)	3 (3.2%)
Dialyses	1 (1.5%)	1 (3.8%)	2 (2.1%)
Macular hole + giant tear	1 (1.5%)	0 (0.0%)	1 (1.1%)
U-tear + macular hole	1 (1.5%)	0 (0.0%)	1 (1.1%)
U-tear + dialyses	1 (1.5%)	0 (0.0%)	1 (1.1%)
U-tear + atrophic hole (peripheral)	6 (8.8%)	2 (7.7%)	8 (8.5%)
Macular hole + atrophic hole (peripheral)	3 (4.4%)	0 (0.0%)	3 (3.2%)
**Total**	**68 (100.0%)**	**26 (100.0%)**	**94 (100.0%)**

**Figure 3:**
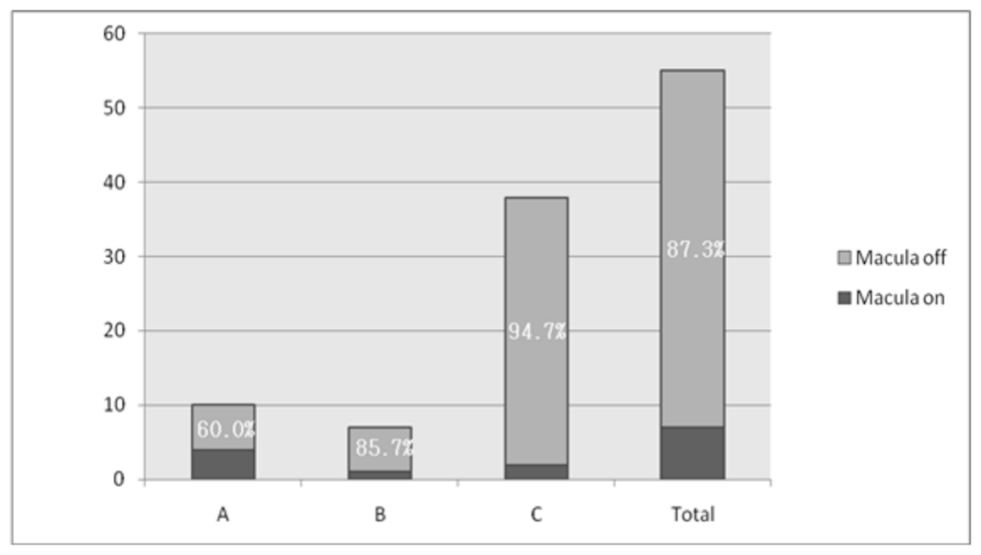
Grade of proliferative vitreoretinopathy (PVR) and macular status of eyes with RRD.

Macula was off in 77 eyes accounting for 81.9%. When considering only RRD (55 eyes), thirty-eight eyes (69.1% of RRD eyes) had PVR of grade C. From RRD eyes, 48 (87.3%) had detached macula. There was a statistically significant association between macular status and PVR status (*P* = 0.011) (Figure 3).

Twenty eyes (36.4% of RRD eyes) had PVR of grade C and all these eyes (100.0%) had the onset of symptoms greater than six months before presentation. There was a statistically significant association between duration of symptoms and grade of PVR (*P* = 0.005) (Table 7). Similarly 20 eyes (36.4% of RRD eyes) had the macula off at presentation and all had the onset of symptoms greater than six months before presentation. When using six months as a cut-off point there was a statistically significant association between duration of symptom and macular status (*P*= 0.040) (RR=1.25 [95% CI; 1.059, 1.475) (Table 8). Sociodemographic characteristics like age, sex, religion, ethnicity, literacy and occupation did not have a statistically significant association with the duration of presentation, macular status and grade of PVR (*P* > 0.05). But family monthly income had a statistically significant association with duration of symptoms (*P* = 0.007 (Table 9).

**Table 7:** Association of duration of symptoms with grade of PVR among eyes with RRD.

**Variable**	**Grade of PVR No (% within duration**)			**Total**
**Duration of symptoms**	**A**	**B**	**C**	**No (%)**
< 1 month	4 (40.0)	0 (0.0)	6 (60.0)	10 (100.0)
1-3 months	4 (22.2)	5 (27.8)	9 (50.0)	18 (100.0)
> 3-6 months	2 (28.6)	2 (28.6)	3 (42.9)	7 (100.0)
> 6 months-1 year	0 (0.0)	0 (0.0)	14 (100.0)	14 (100.0)
> 1 year	0 (0.0)	0 (0.0)	6 (100.0)	6 (100.0)
**Total**	**10 (18.2)**	**7 (12.7)**	**38 (69.1)**	**55 (100.0)**

Abbreviations: PVR, proliferative vitreoretinopathy; RRD, rhegmatogenous retinal detachment

**Table 8:** Association of duration of symptoms with macular status in eyes with RRD.

**Variable**	**Macular status (% within duration)**		**Total**
**Duration of symptoms**	**ON No (%)**	**OFF No (%)**	**No (%)**
< 6 months	7 (20.0)	28 (80.0)	35 (100.0)
≥ 6 months	0 (0.0)	20 (100.0)	20 (100.0)
**Total**	**7 (14.0)**	**48 (86.0)**	**55 (100.0)**

Abbreviation: RRD, rhegmatogenous retinal detachment

**Table 9:** Association of family monthly income with delay in presentation in eyes with all types of RD.

**Variable**	**Duration of symptoms (% within family monthly income)**		**Total**
**Family monthly income (ETB)**	**< 3 months No (%**)	**≥ 3 months No (%**)	**No (%)**
< 500	33 (40.7)	48 (59.3)	81 (100.0)
> 500-1000	1 (14.3)	6 (85.7)	7 (100.0)
> 1000-1500	4 (100.0)	0 (100.0)	4 (100.0)
> 1500-2000	0 (0.0)	0 (0.0)	0 (100.0)
> 2000	2 (100.0)	0 (0.0)	2 (100.0)
**Total**	**40 (42.6)**	**54 (57.4)**	**94 (100.0)**

Abbreviation: ETB, Ethiopian Birr

## Discussion

In this study there were 94 eyes with retinal detachment over a six month period. This is higher than what was reported in a B-scan ultrasonography aided study at Menelik II Hospital [12] which detected 71 RD eyes over same period. This is possibly due to the difference in the socioeconomic characteristics and the study design.

Of 80 RD patients we diagnosed, 58 of them had RRD. A retrospective descriptive study on patients with RD at Retina Clinic of Menelik II Hospital in Addis Ababa from April 1999 to October 2003 (4 years and six months period) showed that there were 305 patients with a diagnosis of RRD [13]. This is estimated to be on average 68 patients in six month period and this is comparable to our finding.

The mean age of our patients was 41.4 years and this is lower than the findings in Minnesota, 54 years [15] and in Norway, 59 years [16]. It is, however, comparable to African studies done in Addis Ababa [13], Zaire [8] and Kenya [17] where the mean age was 41, 40, and 47 years respectively.

Compared to Minnesota [15] and Japanese [18] studies, where trauma was responsible for only 7% and 1.6% of detachments respectively, trauma was the main risk factor for retinal detachment in our study settings accounting for 34.0% of all RD eyes and 45.5% RRD eyes. This is in agreement with other studies in developing nations which showed that ocular trauma was a risk factor for 20.7%-30% of RRD cases [9,13].

In our study, more eyes (41.8% of RRD eyes) had myopia as compared to the study in Addis Ababa [13], where myopia was the predisposing factor for RRD in only 28.3% eyes. Pseudophakia was one of the least important risk factor (10.6%) for RD in our study and this was comparable with the reports in South Africa, 9.6% [9] and in Addis Ababa, 14.2% [13].

In developing countries many patients with RD present late. This study also revealed the same. About 69% of our patients presented at least one month after the onset of symptoms and this is similar to other studies [8,9] which reported a higher figure (62%-70%).

The commonest type of retinal break was U-tear (horse-shoe) which occurred in 20.2% eyes in this study. This figure is comparable to South African [9] study where U-tears were causes for 28% of RD. In Cambridge [19] giant retinal tears caused 1.4% of the entire RD while in Kenya [17]; giant tears were responsible for 8.3% of all RD. In this study, giant tears were present in 4.3%.

Macula was off in 81.9% eyes which is higher than the finding in a UK study (58.8%) [20], but comparable to the report in Iran (81.6%) [21], India (86.9%) [14] and Kenya (91.9%) [17]. It is also slightly higher than the Menelik II study where 73.8% of the eyes’ macula was off (13). This could be due to delayed presentation of patients and this is supported by the statistically significant association between macular status and time of presentation shown in this study.

We found a higher percent (73.4%) of grade C PVR as compared to the 4% in Cambridge (19), 13% in Iranian (21), 18% in Kenyan (17), 18.7% in Addis Ababa (13) and 33% in South African (9) studies. This difference could be explained by the late presentation of our patients as grade of PVR was significantly associated with delay in presentation. We also found a higher number (10.6%) of macular holes in this study in comparison to the study in Helsinki (19), where only 1.7% of RD had a macular hole.

Though this study is the first comprehensive prospective study in southwest Ethiopia and valuable for understanding the epidemiology of RD among blacks and establishing a vitreoretinal service in the region, it has its own limitations. First, it was a hospital-based study so the data generated cannot be accurately extrapolated to the general population. Second, A/B-Scan Ultrasound was not used and thus we excluded patients with dense ocular media opacity which could underestimate the magnitude of retinal detachment in the study setting.

In conclusion, we demonstrated that a significant numbers of patients with ocular problem have retinal detachment, and retinal detachment affected wide range of age groups and more of male adults. Trauma and myopia were the most important risk factors for retinal detachment. Most of the patients presented late and had high-grade PVR. Eye care workers should give education on eye safety precautions and prevention of ocular traumas. We recommend a population based study to assess the epidemiology and risk factors and other socioeconomic factors affecting the health seeking behavior and late presentation.
