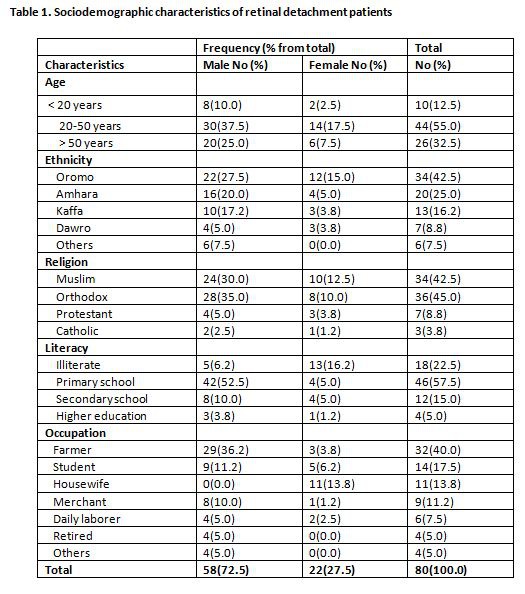# Correction: Retinal Detachment in Southwest Ethiopia: A Hospital Based Prospective Study

**DOI:** 10.1371/annotation/2b8e3b87-642d-4f8a-8810-2161ad74f730

**Published:** 2013-10-25

**Authors:** Tsedeke Asaminew, Yeshigeta Gelaw, Sisay Bekele, Berhan Solomon

There was an error in Table 1. The correct version of the table is available here: 

**Figure:**